# Association of six CpG-SNPs in the inflammation-related genes with coronary heart disease

**DOI:** 10.1186/s40246-016-0067-1

**Published:** 2016-07-25

**Authors:** Xiaomin Chen, Xiaoying Chen, Yan Xu, William Yang, Nan Wu, Huadan Ye, Jack Y. Yang, Qingxiao Hong, Yanfei Xin, Mary Qu Yang, Youping Deng, Shiwei Duan

**Affiliations:** 1 Cardiovascular Center of Ningbo First Hospital, Ningbo University, Ningbo, Zhejiang 315010 China; 2 Texas Advanced Computing Center, University of Texas at Austin, 10100 Burnet Road (R8700), Austin, TX 78758-4497 USA; 3 School of Medicine, Ningbo University, Ningbo, Zhejiang 315211 China; 4 Center of Safety Evaluation, Zhejiang Academy of Medical Sciences, Hangzhou, Zhejiang 310007 China; 5 MidSouth Bioinformatics Center, Department of Information Science, George Washington Donaghey College of Engineering and Information Science, and Joint Bioinformatics Graduate Program, University of Arkansas at Little Rock and University of Arkansas for Medical Sciences, 2881 S. University Ave, Little Rock, AR 72204 USA; 6 Medical College, Wuhan University of Science and Technology, Wuhan, 430064 China; 7 Department of Internal Medicine and Biochemistry, Rush University Medical Center, Chicago, IL 60612 USA

**Keywords:** Coronary heart disease, Inflammation, Promoter, CpG-SNP, Polymorphism

## Abstract

**Background:**

Chronic inflammation has been widely considered to be the major risk factor of coronary heart disease (CHD). The goal of our study was to explore the possible association with CHD for inflammation-related single nucleotide polymorphisms (SNPs) involved in cytosine-phosphate-guanine (CpG) dinucleotides. A total of 784 CHD patients and 739 non-CHD controls were recruited from Zhejiang Province, China. Using the Sequenom MassARRAY platform, we measured the genotypes of six inflammation-related CpG-SNPs, including *IL1B* rs16944, *IL1R2* rs2071008, *PLA2G7* rs9395208, *FAM5C* rs12732361, *CD40* rs1800686, and *CD36* rs2065666). Allele and genotype frequencies were compared between CHD and non-CHD individuals using the CLUMP22 software with 10,000 Monte Carlo simulations.

**Results:**

Allelic tests showed that *PLA2G7* rs9395208 and *CD40* rs1800686 were significantly associated with CHD. Moreover, *IL1B* rs16944, *PLA2G7* rs9395208, and *CD40* rs1800686 were shown to be associated with CHD under the dominant model. Further gender-based subgroup tests showed that one SNP (*CD40* rs1800686) and two SNPs (*FAM5C* rs12732361 and *CD36* rs2065666) were associated with CHD in females and males, respectively. And the age-based subgroup tests indicated that *PLA2G7* rs9395208, *IL1B* rs16944, and *CD40* rs1800686 were associated with CHD among individuals younger than 55, younger than 65, and over 65, respectively.

**Conclusions:**

In conclusion, all the six inflammation-related CpG-SNPs (rs16944, rs2071008, rs12732361, rs2065666, rs9395208, and rs1800686) were associated with CHD in the combined or subgroup tests, suggesting an important role of inflammation in the risk of CHD.

## Background

Coronary heart disease (CHD) is considered to be the leading cause of mortality and morbidity in the elderly [1]. CHD has emerged as a serious burden to human health [2]. Both genetic and environmental factors were shown to play an important role in the development of CHD [3]. Environmental factors, including smoking and dietary changes, were found to be associated with CHD [4]. Heritable factors were estimated to contribute to 30–60 % of the variation in the risk of CHD [3]. In addition, the incidence of CHD was generally higher in men than in women regardless of their menopause type [5]. Therefore, further exploration of the interactive mechanism between genes and environment is much more significant and helpful for specific diagnosis [6].

Increasing amount of evidence has indicated that inflammation is responsible for CHD [7, 8]. Many inflammation-related genes are found to be associated with risk of CHD [9, 10]. Interleukin 1, beta gene (*IL1B*) encodes the cytokine which exerts a wide range of inflammatory activities [11]. Genetic association between *IL1B* and CHD has been previously found [12]. Interleukin 1 receptor, type II (*IL1R2*) encodes a cytokine receptor that belongs to the interleukin 1 receptor family [13]. Notably, *IL1R2* is recognized as a general factor in inflammatory response [14]. Phospholipase A2, group VII (*PLA2G7*) gene encodes a kind of secreted enzyme [15], and patients with high *PLA2G7* expression was found to have an increased risk of cardiovascular diseases [16]. Gene polymorphisms in family with sequence similarity 5, member C (*FAM5C*) gene were shown to be associated with an increased risk of acute myocardial infarction [17], and elevated FAM5C levels were found in atherosclerotic plaques and coronary artery endothelium [18]. *CD40* encodes a cell surface receptor that plays a pivotal role in macrophage activation and parasite immunity [19], and *CD40* could up-regulate prime macrophages through a proinflammatory program in recent studies [19]. *CD36* encodes a platelet receptor glycoprotein involved in different biological processes such as inflammation, atherosclerosis, and platelet activation [20]. The association between monocyte/macrophage *CD36* and atherosclerosis has been found in the previous studies [21].

Single nucleotide polymorphisms (SNPs) can change biological properties of the encoded protein and affect gene expression levels in an allele-specific manner (24450106). Due to the mutability of cytosine-phosphate-guanine (CpG) dinucleotides, CpG-SNPs, as an important class of cis-regulatory polymorphisms, connect genetic variation to the individual variability of the epigenome [22]. CpG-SNPs in the promoter regions had been found to be associated with multiple diseases, including type 2 diabetes [23], breast cancer [24], schizophrenia [25], and epithelial ovarian cancer [26]. In light of previous studies, we aimed to evaluate whether the six inflammation-related gene CpG-SNPs could contribute to the risk of CHD.

## Results and discussion

Genotypic and allelic tests were performed for a total of six CpG-SNPs between CHD and non-CHD individuals (Table 1). According to the most recent human assembly, hg38/GRCh38, five CpG-SNPs (*FAM5C* rs12732361, *IL1R2* rs2071008, *IL1B* rs16944, *CD40* rs1800686, and *CD36* rs2065666) were located upstream of the corresponding transcription start sites (47, 178, 387, 508, and 659 bp, respectively). The remaining one (*PLA2G7* rs9395208) was located in the 111 bp downstream of transcription start sites.

**Table 1:** The list of genotyping primers for six CpG-SNPs

Gene	SNP	Primers	Sequence (5′ to 3′)
*IL1B*	rs16944	1st PCR primer	ACGTTGGATGAGAGGCTCCTGCAATTGACA
		2nd primer	ACGTTGGATGCTGTCTGTATTGAGGGTGTG
		Extend primer	GGGGTGGGTGCTGTTCTCTGCCTC
*IL1R2*	rs2071008	1st PCR primer	ACGTTGGATGGAAAAATCCATGCAGCCTCC
		2nd primer	ACGTTGGATGTGGTGGCTGACTTTCCAAGG
		Extend primer	TGGGAAGAAGCAAGCACCCC
*PLA2G7*	rs9395208	1st PCR primer	ACGTTGGATGTGGACCCGCGGTTAACTTAG
		2nd primer	ACGTTGGATGATCAGGTCTGCGGAAAGGAG
		Extend primer	GCATTGCCTGGCTCT
*FAM5C*	rs12732361	1st PCR primer	ACGTTGGATGTTACACAGAGAGCCACGAAC
		2nd primer	ACGTTGGATGAGGATCACCACGAATCACCC
		Extend primer	GAAACCCCCACCATTCCCCA
*CD40*	rs1800686	1st PCR primer	ACGTTGGATGATGGATGGGAAGTTGAGACG
		2nd primer	ACGTTGGATGCCCAACTCAGAATTTCGCTC
		Extend primer	GTCGCTTTCAAAGGAAATTCCCT
*CD36*	rs2065666	1st PCR primer	ACGTTGGATGCTCTGAAGATATAATGACAAG
		2nd primer	ACGTTGGATGCAGTTTCTCTGTTCACTTCG
		Extend primer	CGTTCACTTCGTTTTAGTATAGAATTA

As shown in Table 2, the genotype distributions of all the six SNPs in the non-CHD controls met Hardy-Weinberg equilibrium (HWE). Our results showed that *PLA2G7* rs9395208 and *CD40* rs1800686 were significantly associated with CHD on the allele level. The frequency of allele rs9395208-G was significantly lower in the case group than in the control (84.7 versus 87.3 %; *P* = 0.04, OR = 0.806, 95 % CI = 0.657–0.991). Meanwhile, a higher rs1800686-G frequency showed in the cases than in controls (69.4 versus 65.4 %; *P* = 0.02; OR = 1.198, 95 % CI = 1.029–0.394). No significant difference was observed on the genotype level for all the CpG-SNPs (*P* > 0.05).

**Table 2:** Comparisons of genotype and allele frequencies between cases and controls

SNP	Groups	Genotype (counts)			*χ* ^2^	*P* (*df* = 2)	HWE *P* value^a^	Allele (counts)		*χ* ^2^	*P* (*df* = 1)	OR (95 % CI)
*IL1B* rs16944		GG	AG	AA				G	A			
	Cases	220	380	176			0.627	820	732			
	Controls	173	379	180	4.387	0.112	0.335	725	739	3.310	0.069	1.142 (0.990–1.317)
*IL1R2* rs2071008		GG	GT	TT				G	T			
	Cases	434	285	65			0.064	1153	415			
	Controls	417	275	46	2.382	0.304	0.941	1109	367	1.020	0.313	0.919 (0.781–1.082)
*PLA2G7* rs9395208		GG	GC	CC				G	C			
	Cases	565	198	21			0.468	1328	240			
	Controls	568	154	17	4.603	0.100	0.095	1290	188	4.210	0.040	0.806 (0.657–0.991)
*FAM5C* rs12732361		GG	AG	AA				G	A			
	Cases	492	242	49			0.011	1226	340			
	Controls	431	254	52	3.022	0.221	0.088	1116	358	2.850	0.091	1.157 (0.977–1.370)
*CD40* rs1800686		GG	AG	AA				G	A			
	Cases	378	332	74			0.929	1088	480			
	Controls	317	333	89	5.411	0.067	0.914	967	511	5.440	0.020	1.198 (1.029–1.394)
*CD36* rs2065666		GG	GC	CC				G	C			
	Cases	439	288	56			0.356	1166	400			
	Controls	411	282	43	1.24	0.538	0.555	1104	368	0.120	0.729	0.972 (0.825–1.145)

^a^ *HWE* Hardy-Weinberg equilibrium; *P* value <0.05 was considered a departure from HWE

Gender disparities widely existed in the prevalence of CHD [27], and female CHD patients aged 40 or older have 10 % greater risk of death than the males [27]. Hence, we performed a gender-based subgroup analysis to detect the difference both in genotype and allele frequencies (Table 3). It showed significant associations of *FAM5C* rs12732361 and *CD36* rs2065666 with CHD in males on the allele level (*P* = 0.03, OR = 1.266, 95 % CI = 1.022–1.568; *P* = 0.04, OR = 0.806, 95 % CI = 0.652–0.996, respectively). Meanwhile, *CD40* rs1800686 was shown to be significantly associated with CHD in females (*P* = 0.02, OR = 1.347, 95 % CI = 1.043–1.740 by allele).

**Table 3:** A list of SNPs associated with CHD in the subgroup tests

Gene	SNP	Model or subgroup	Group	Genotype or allele (counts)	*χ* ^2^	P (*df*)	OR (95 % CI)
*IL1B*	rs16944	Dominant	Cases	GG/GA + AA (220/556)	4.350	0.037 (1)	1.279 (1.015–1.611)
			Controls	GG/GA + AA (173/559)			
*IL1B*	rs16944	≤55 years of age	Cases	G/A (236/236)	6.190	0.013 (1)	1.422 (1.077–1.877)
			Controls	G/A (292/248)			
*IL1B*	rs16944	55–65 years of age	Cases	GG/GA/AA (79/134/57)	6.147	0.046 (2)	
			Controls	GG/GA/AA (53/145/66)			
*IL1B*	rs16944	55–65 years of age	Cases	G/A (292/248)	4.560	0.033 (1)	1.299 (1.022–1.653)
			Cases	G/A (251/277)			
*IL1R2*	rs2071008	≤55 years of age	Controls	G/T (254/106)	3.860	0.049 (1)	0.733 (0.538–1.000)
			Cases	G/T (366/112)			
*PLA2G7*	rs9395208	Dominant	Cases	GG/GC + CC (565/219)	4.590	0.032 (1)	0.777 (0.616–0.979)
			Controls	GG/GC + CC (568/171)			
*PLA2G7*	rs9395208	≤55 years of age	Cases	G/C (295/65)	5.750	0.016 (1)	0.627 (0.427–0.920)
			Controls	G/C (420/58)			
*FAM5C*	rs12732361	Male	Cases	G/C (849/225)	4.660	0.031 (1)	1.266 (1.022–1.568)
			Controls	G/C (629/211)			
*CD40*	rs1800686	Female	Cases	G/A (354/138)	5.230	0.022 (1)	1.347 (1.043–1.740)
			Controls	G/A (417/219)			
*CD40*	rs1800686	Dominant	Cases	GG/GA + AA (378/406)	4.340	0.037 (1)	1.239 (1.013–1.517)
			Controls	GG/GA + AA (317/422)			
*CD40*	rs1800686	≥65 years of age	Cases	GG/GA/AA (163/146/24)	9.437	0.009 (2)	
			Controls	GG/GA/AA (90/109/32)			
*CD40*	rs1800686	≥65 years of age	Cases	G/A (472/194)	8.600	0.0034 (1)	1.456 (1.132–1.874)
			Controls	G/A (289/173)			
*CD36*	rs2065666	Male	Cases	G/C (790/286)	4.000	0.046 (1)	0.806 (0.652–0.996)
			Controls	G/C (641/187)			

Age is another well-known risk factor in the development and prognosis of CHD [28], and there were over 70 % of coronary-related deaths occurred in the people older than 70 in North America and Western Europe [28]. Therefore, we further evaluated the genotype and allele frequencies in different age subgroups (Table 3). For the individuals younger than 55, the frequencies of both rs16944-G and rs2071008-G alleles were significantly lower in case group than in control group (*P* = 0.01, OR = 1.422, 95 % CI = 1.077–1.877; *P* = 0.04, OR = 0.733, 95 % CI = 0.538–1.000, respectively). For the individuals with age between 55 and 65 years old, rs16944 was associated with CHD on both genotype and allele levels (*χ*^2^ = 6.15, *P* = 0.04 by genotype; *P* = 0.03, OR = 1.299, 95 % CI = 1.022–1.653 by allele). Among the individuals older than 55, rs1800686 was also shown to be associated with CHD on both genotype and allele levels (genotype: *χ*^2^ = 9.44, *P* = 0.009; allele: *P* = 0.003; OR = 1.456, 95 % CI = 1.132–1.8740).

Additionally, we have conducted a comparison under the dominant and recessive inheritance models between cases and controls (Table 3). In the dominant model, significant associations among rs16944, rs9395208 and rs1800686 with CHD were observed. The rs16944-A and rs1800686-A alleles were risk factors for CHD (rs16944: *P* = 0.03, OR = 1.279, 95 % CI = 1.015–1.611; rs1800686: *P* = 0.04, OR = 1.239, 95 % CI = 1.013–1.517). The rs9395208-C allele was a protective factor for CHD (*P* = 0.03, OR = 0.777, 95 % CI = 0.616–0.979). Meanwhile, all the six CpG-SNPs showed no significant association with CHD in the recessive model.

Besides, a post hoc power analysis showed that our association study had strong power (93.9–94.1 %) to detect significant association of the six CpG-SNPs under an OR of 1.3.

The present study performed a comprehensive analysis of association between six CpG-SNPs (*IL1B* rs16944, *IL1R2* rs2071008, *PLA2G7* rs9395208, *FAM5C* rs12732361, *CD40* rs1800686, and *CD36* rs2065666) and CHD. At the allelic level, *CD40* rs1800686-G was found to be a risk factor, while *PLA2G7* rs9395208-G was found to be a protective factor. Moreover, all the CpG-SNPs were significantly associated with the risk of CHD in the combined or subgroup analyses.

Inflammation represents an important feature in the process of atherosclerosis, which can form, destabilize, and rupture atherosclerotic plaques, finally causing CHD [29]. Over the years, researchers have found some inflammatory factors related to the pathogenesis and prognosis of CHD, such as *CLOCK* SNP rs4580704 [30], *ICAM-1* SNP rs281432 [31], and *NFKB1* SNP rs28362491 [32]. Some of these inflammation-related genes were expected to be drug targets for the control and treatment of CHD. Meanwhile, we hypothesized that hereditable methylation could be associated with CHD. Because of low minor allele frequency or weak haplotype associations, genome-wide searches for genetic risk factors for CHD have in general not investigated the CpG-SNPs. Our previous work [33] indicated that CpG-SNPs of the thrombotic pathway genes contributed to the risk of CHD, which suggested a clue for investigating the contributions of the inflammation-related CpG-SNPs to the susceptibility to CHD.

*PLA2G7* gene encodes a secreted enzyme whose activity is associated with CHD [34]. *PLA2G7* functions as a biomarker of plaque inflammation and stability [35]. Several *PLA2G7* SNPs (e.g., rs7756935, rs1805017, and rs13210554) have been reported on the susceptibility to CHD [35, 36]. Here, we discovered a significant association of *PLA2G7* CpG-SNP rs9395208 with CHD, even in the individuals aged ≤55, providing additional evidence of age dimorphism in the risk of CHD. Additionally, Jiang et al. [15] have reported that the correlation between *PLA2G7* methylation and CHD risk in females is independent of age, smoking, diabetes, and hypertension, which indicated a close relationship between *PLA2G7* and hereditable methylation in the pathogenic mechanism of CHD.

The contribution of *CD40* rs1800686 is another main finding in the current study. *CD40* is considered to determine T cell responses to antigen presentation and B cells immunoglobulin isotype switching, which plays a key role in the inflammatory and prothrombotic processes by bonding with CD40 ligand (CD40L) in atherosclerosis [37]. Previous study provided evidence of association of *CD40* rs1883832 with an overall increased risk of CHD in Chinese population [38]. In the current study, a similar result was drawn that *CD40* rs1800686-A allele contributed to CHD risk. Age and gender are considered as two independent risk factors in CHD [28, 39]. Subsequent subgroup analysis identified age and gender differences existed in the rs1800686, which revealed an age- and gender-based mechanism on the genetic variations.

As a scavenger receptor, CD36 is not only involved in the metabolism of lipids but also plays an important role in the adhesion of negatively charged macromolecules [40]. CD36 is widely expressed in cells and tissues including microvascular endothelial cells, monocytes, and macrophages [41]. Previous study implied that increased CD36 expression level could reflect the severity of coronary artery atherosclerosis [42]. Several *CD36* SNPs (e.g., rs5956, rs3173798, and rs3211892) have been reported to be associated with CHD [42]. Here, we detected a significant protection role of *CD36* CpG-SNP rs2065666 G carries in CHD male patients. Our data suggested a possible molecular mechanism through which SNP could influence a phenotype.

Interleukins (IL-1B and IL1R2) were shown to play a role in the inflammatory response, and they lead to the development of atherosclerotic plaques [43, 44]. Recently, gender dimorphism was observed in the association of *IL-1B* polymorphism with CHD [45]. We find no significant difference on *IL-1B* rs16944 allele or genotype level by gender subgroup analysis. However, age-based subgroup tests showed that *IL-1B* rs16944-G was a risk factor of CHD in younger population, while *IL1R2* rs2071008-G was a protective factor. Further validation study or functional analysis of these variants is needed in the future.

Increased expression of FAM5C may be induced by inflammatory stimuli [17]. This case-control study observed significant associations between *FAM5C* rs12732361 polymorphisms and CHD only in males. Due to a lack of CHD study on *FAM5C*, more investigations are needed to validate our results.

There are also limitations in the current study. The findings of our study are limited to a small population with documented CHD and cannot be generalized to the population at large. Although we found positive results in the current study, the power effect might be reduced by strict multiple adjustment or subgroup analyses. Therefore, larger sample size and other ethnic populations are required to be investigated.

## Conclusions

In conclusion, our case-control study suggested that the six inflammation-related CpG-SNPs were significantly associated with the risk of CHD in the combined or the subgroup samples. Moreover, our results also revealed that *IL1B* rs16944, *PLA2G7* rs9395208, and *CD40* rs1800686 had a significant contribution to the risk of CHD under the dominant model.

## Methods

### Sample collection

A total of 784 CHD patients and 739 healthy controls were collected from Ningbo First Hospital of Ningbo University between May 2008 and April 2015 in Zhejiang province, China. CHD patients and non-CHD controls were defined as described before [33, 46]. Patients with congenital heart disease, cardiomyopathy, liver disease, and renal disease were excluded. Blood samples were collected in 3.2 % citrate sodium-treated tubes and processed in the central clinical laboratory of the hospital. All the participants provided written informed consent form under a protocol approved by the Medical Ethics Committees of Ningbo First Hospital and Ningbo University.

### SNP selection and genotyping

The selected CpG-SNPs are on the promoter of inflammation-related genes. Meanwhile, the minor allele frequencies of the selected CpG-SNPs are over 10 % in HapMap HCB population. CpG-SNPs with design problems or failed assays were excluded. Finally, six CpG-SNPs in inflammation-related genes were included in the current study. DNA extraction and quantification were described as previously [33, 46]. The polymerase chain reaction (PCR) amplification was performed on the GeneAmp® PCR System 9700 (Applied Biosystems, Foster City, CA, USA), and the genotyping was performed on the MassARRAY iPLEX® assay platform (Sequenom, San Diego, CA, USA). The sequences of the amplification and extension primers for the six CpG-SNPs were shown in Table 1.

### Statistical analyses

Genotype and allele frequencies of the polymorphisms between cases and controls were calculated by the CLUMP22 software with 10,000 Monte Carlo simulations [47]. The distribution of Hardy-Weinberg equilibrium (HWE) was tested using the Arlequin program (version 3.5, Bern, Switzerland), and *P* > 0.05 was considered to be in HWE. Power analysis was performed using the Power and Sample Size Calculation software (v3.0.43). A two-tailed *P* < 0.05 was considered statistically significant.
